# Lyssavirus matrix protein cooperates with phosphoprotein to modulate the Jak-Stat pathway

**DOI:** 10.1038/s41598-019-48507-4

**Published:** 2019-08-21

**Authors:** Florian Sonthonnax, Benoit Besson, Emilie Bonnaud, Grégory Jouvion, David Merino, Florence Larrous, Hervé Bourhy

**Affiliations:** 1 Unité Lyssavirus, Epidémiologie et Neuropathologie, Institut Pasteur, 28, rue du Docteur Roux, 75724 Paris, Cedex 15 France; 2 Université Paris-Diderot, Sorbonne-Paris Cité, Cellule Pasteur, rue du Docteur Roux, 75724 Paris, Cedex 15 France; 3 Unité de Neuropathologie expérimentale, Institut Pasteur, 28, rue du Docteur Roux, 75724 Paris, Cedex 15 France

**Keywords:** Viral pathogenesis, Virus-host interactions, Viral immune evasion

## Abstract

Phosphoprotein (P) and matrix protein (M) cooperate to undermine the immune response to rabies virus (RABV) infections. While P is involved in the modulation of the Jak-Stat pathway through the cytoplasmic retention of interferon (IFN)-activated STAT1 (pSTAT1), M interacts with the RelAp43-p105-ABIN2-TPL2 complex, to efficiently inhibit the nuclear factor-κB (NF-κB) pathway. Using transfections, protein-complementation assays, reverse genetics and DNA ChIP, we identified a role of M protein in the control of Jak-Stat signaling pathway, in synergy with the P protein. In unstimulated cells, both M and P proteins were found to interact with JAK1. Upon type-I IFN stimulation, the M switches toward pSTAT1 interaction, which results in an enhanced capacity of P protein to interact with pSTAT1 and restrain it in the cytoplasm. Furthermore, the role for M-protein positions 77, 100, 104 and 110 was also demonstrated in interaction with both JAK1 and pY-STAT1, and confirmed *in vivo*. Together, these data indicate that M protein cooperates with P protein to restrain in parallel, and sequentially, NF-κB and Jak-Stat pathways.

## Introduction

Rabies virus (RABV), a neurotropic virus belonging to the Lyssavirus genus of the *Rhabdoviridae* family, causes an invariably fatal encephalitis in humans and animals^1^. Prophylaxis is highly effective but control of the disease in developing countries is difficult due to economic and political factors. Hence, this zoonotic infection is responsible for about 60,000 deaths/year, mainly in Asia and Africa^2^.

The principal host-cell response to viral infections is activation of the innate immune response mediated by type-I interferon (IFNα/β)^3^. Following cell infection, viral RNA is detected by receptors (e.g. retinoic acid-inducible gene I (*RIG-I*)), which activate several signaling pathways, including through the nuclear factor-kappa B (NF-κB) family proteins. NF-κB dimers translocate into the nucleus and activate specific genes under the regulation of κB promoters, including tripartite motif protein-25 (*TRIM25*) and *IFN*α*/*β. Subsequent binding of IFNα/β to IFNα/β receptor 1 (IFNAR1) and IFNAR2C activates Janus kinase-1 (JAK1) and tyrosine kinase-2 (TYK2). Signal transducers and activators of transcription (STAT)-1 and -2 are then phosphorylated at residues Y701 and Y690, respectively. Dimers of activated STAT1/2 interact with IFN-response factor-9 (IRF9) to form the IFN-stimulated gene factor-3 (ISGF3) complex, which translocates to the nucleus to activate IFN-stimulated genes (*ISG*s) regulated by IFN-stimulated response-element (ISRE) promoters, including *ISG*1*5* and *MxA*^4^.

Viruses have evolved powerful countermeasures to evade host innate immunity^5^. Some viral proteins act as NF-κB pathway inhibitors, including vaccinia virus K1 protein^6^ and human immunodeficiency virus Vpu protein^7^. STAT proteins are major targets of many viral IFN antagonists, including those of measles virus (V protein)^8^, Sendai virus (C protein)^9^, dengue virus (NS5 protein)^10^ and influenza virus (NS1 protein)^11^. Polyomavirus large T^12^ inhibits signal transduction through JAK1, and Marburg virus matrix protein VP40^13,14^ targets JAK1 to inhibit its phosphorylation and the subsequent activation of STAT1/2.

RABV encodes only five proteins (nucleoprotein (N), phosphoprotein (P), matrix protein (M), glycoprotein (G) and viral polymerase (L)) but is able to potentialy subvert the biology of infected cells. This derives from the multifunctional nature of several of the proteins including P and M proteins, which, other than conserved roles in viral replication and assembly, are both involved in evading the host’s innate immune responses through regulation of cell signaling. Lyssavirus interference with the Jak–Stat pathway has been extensively characterized over more than a decade^15,16^, including the roles of P protein and its isoforms in the cytoplasmic retention of STAT proteins. The P-protein C-terminal domain and particularly residues W265 and M287 are involved in the interaction with IFN-activated STAT1 (pSTAT1) and cytoplasmic retention of pSTAT1 to evade the host innate immunity^17,18^. M protein and particularly residues R77, D100, A104 and M110 plays a pivotal role in immune-response inhibition by targeting a NF-κB complex involving RelAp43, a splice variant of RelA(p65)^19,20^.

Herein, we used an RABV street isolate from a dog in Thailand (Tha) that is representative of currently circulating RABVs^21^, to study the effects of M and/or P proteins on the Jak-Stat pathway. Based on previous results^17,18,22^, we introduced mutations into Tha-virus P and M proteins. Notably, we showed that M protein also interacted with pSTAT1 to induce the latter’s cytoplasmic retention, and with JAK1 to inhibit its activation. *In vivo* mouse studies showed that mutated M protein attenuated virulence and increased anti-viral immune responses. Thus, M protein’s role is crucial and not restricted to only NF-κB–pathway inhibition, but extends to inhibition of the Jak-Stat-pathway. Taken together, those findings reinforce the idea that RABV proteins have evolved to cooperate in neutralization of the innate immune response.

## Results

### Mutations of RABV P and M proteins result in IFN-pathway stimulation

To examine the effect of Tha viral proteins on the innate immune response, we inserted W265G and/or M287V mutations into P protein and R77K/D100A/A104S/M110L mutations into M protein, to obtain the following mutated RABVs: Th2P (P-W265G/M287V), ThP_265_ (P-W265G), ThP_287_ (P-M287V), Th4M (M-R77K/D100A/A104S/M110L) and Th2P-4M (P-W265G/M287V and M-R77K/D100A/A104S/M110L). In IFN response defective BSR-T7 cells^23^ (Fig. S1A), replication of RABV strains were not impaired and no significant difference of growth was observed in comparison with wild-type Tha. All recombinant RABVs produced similar viral protein levels except the Th2P mutants, which P protein expression was slightly affected (Fig. S1B). In comparison, Th2P, Th4M and Th2P-4M replication was impaired compaired to single ThP mutants and Tha in IFN-competent HeLa cells (Fig. S1C). The heightened sensitivity of Th2P, Th4M and Th2P-4M in IFN stimulated cells indicates intrinsic sensitivity to the IFNα activated Jak-Stat-pathway.

**Figure 1:**
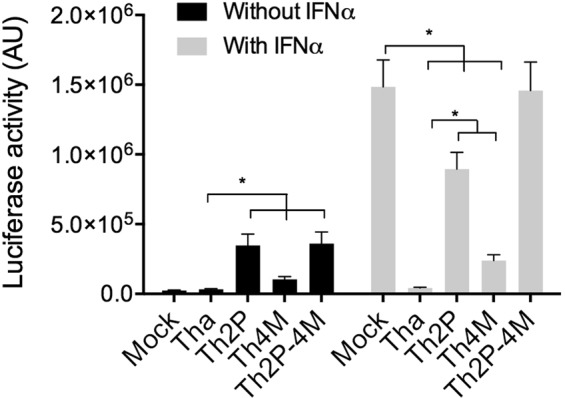
RABVs with mutated P and/or M proteins display enhanced activation of the IFN-pathway compared with wild-type RABV. STING37 cells, which are stably transfected with ISRE promoter-dependent luciferase reporter gene, were infected with wild-type (Tha) RABV or RABV mutated for P- and/or M-mutated protein (Th2P, Th4M, Th2P-4M) RABVs. IFNα (1000 U/mL, 24 h) was added (grey) or not (black) 24 h post-infection. Two days post-transfection, firefly luciferase activity, which is indicative of ISRE-promoter activity, was determined. Results are expressed as the means ± standard deviation (T-bars) of three independent experiments. *p < 0.05.

STING37 cells expressing luciferase under the control of an ISRE promoter were used to quantify the activation of the Jak–Stat-pathway under infection (Fig. 1). Inhibition of ISRE-promoter activation observed in Tha-infected cells was lost in Th2P-infected cells (15 fold increase), confirming that P residues W265 and M287 inhibit Jak–Stat signaling. Surprisingly, the ISRE promoter was also activated (4 times) in Th4M- compared to Tha-virus–infected cells. Therefore, M protein residues R77, D100, A104 and M110 appear to be involved in Jak-Stat-signaling inhibition.

In order to confirm that Th4M modulates directly the Jak-Stat pathway, we bypassed the NF-κB pathway stimulating the cells with IFNα (Fig. 1) In presence of IFNα, only Tha virus controlled the activation the ISRE promoter while Th2P- (20-fold) or Th4M- (6-fold) allowed its activation. These data supported a direct involvement of P and M proteins in inhibition of the Jak-Stat-pathway, and indicated that P protein had a greater impact than M protein. Of note, mutation of both proteins (Th2P-4M) were needed to observe IFNα-dependent activation of the ISRE promoter to a similar extent as that observed for IFNα-treated non-infected cells, indicating that the Jak-Stat-pathway is controlled through combined activity of P-and-M-proteins.

### P and M proteins cooperate in pSTAT1 interactions

**Figure 2:**
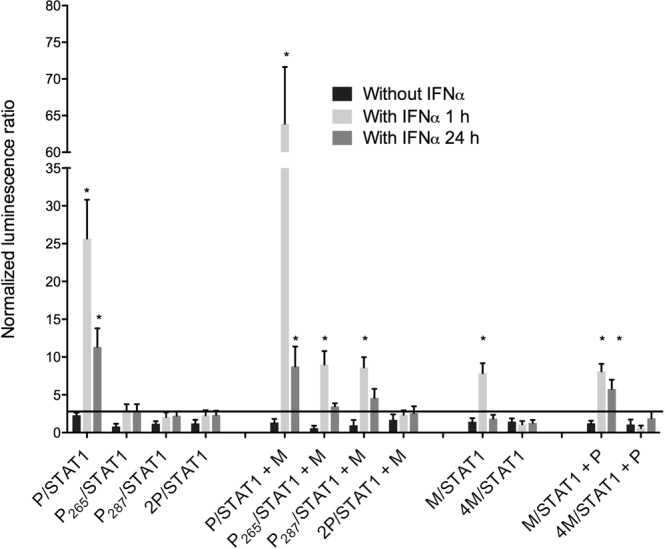
RABV P and/or M proteins cooperate in pSTAT1 interactions. Protein complementation assay: wild-type RABV or RABV with mutated P (P_265_, P_287_, 2P) or M (4M) proteins were co-transfected with STAT1-coding plasmid and stimulated for 1 or 24 h with or without IFNα. Two days post-transfection, *Gaussia* luciferase activity, whose intensity is indicative of interaction of the proteins, was determined. P and M proteins were also transfected as third partners in some experiments. All results are expressed as the means ± standard deviation (T-bars) of at least four independent experiments. *p < 0.05 compared to unstimulated samples.

To decipher the mechanism of Jak-Stat-pathway inhibition, a PCA based on split *Gaussia* luciferase was used. HEK-293T cells were transfected with P, P_265_ (W265G), P_287_ (M287V), 2P (W265G/M287V)^18^, M and 4M (R77K/D100A/A104S/M110L)^22^ and Jak-Stat-signaling proteins, including STAT1 and JAK1 (Fig. 2). An IFNα treatment of 1 or 24 h allowed to distinguish the early and late stages of Jak–Stat-pathway activation, respectively.

As expected, after 1 h of IFNα stimulation, Tha-virus P protein interacted with pSTAT1 and W265G/M287V mutations abrogated that interaction. Co-transfection revealed that M protein stabilized the P/pSTAT1 complex (2.5-fold) and allowed P_265_ and P_287_ interaction with pSTAT1, but not 2P. Therefore, M protein seemed to counterbalance single P-protein mutations (Fig. 2). To a lesser extent, this mechanism was also observable after 24 h of IFNα stimulation. M protein interacted also with pSTAT1 after 1 h of IFNα and required P protein co-transfection to stabilize the interaction after 24 h of treatment. The 4M mutant did not interact with pSTAT1, indicating key roles of mutations R77K/D100A/A104S/M110L in that interaction.

### P and M proteins cooperate in pSTAT1 cytoplasmic retention

**Figure 3:**
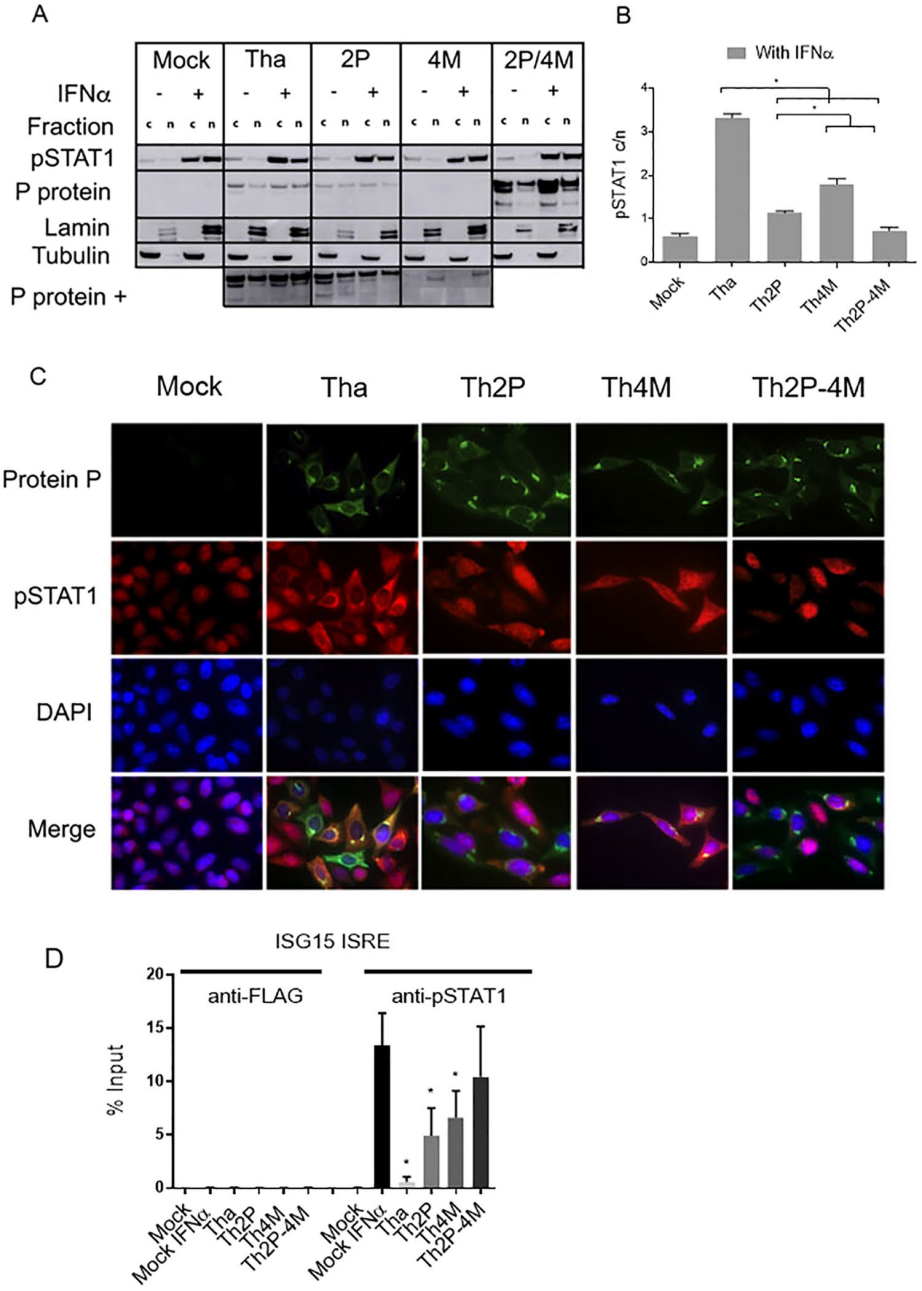
RABV P and/or M proteins are involved in pSTAT1 cytoplasmic retention. (**A**) After cytoplasmic and nuclear fractionation of HeLa cells infected with wild-type (Tha) or P- and/or M-mutated–protein RABVs (Th2P, Th4M, Th2P-4M) and stimulated with IFNα (1000 U/mL, 24 h) or not, the presence of P and pSTAT1 protein was analysed by immunoblotting with specific antibodies. Lamin and tubulin served as controls. Abbreviations, c: cytoplasmic fraction, n: nuclear fraction. (**B**) pSTAT1 cytoplasm/nucleus quantification based on three independent experiments, expressed as means ± standard deviation (T-bars)). *p < 0.05. (**C**) Specific antibody immunolabeling of pSTAT1, and P and M proteins in cells infected with different recombinant RABVs. DAPI was used to stain nuclei blue. (**D**) HeLa cells were infected with wild-type (Tha) or Th2P, Th4M, Th2P-4M. After 24 h of infection, IFNα (1000 U/mL, 24 h) was added. ChIP assays were run with the indicated antibodies. ChIP DNA was analyzed by qPCR by using ISG15-ISRE primers. Results are expressed as the means ± standard deviation (T-bars) of three independent experiments. *p < 0.05.

The effect of P and M protein interactions on STAT1 cellular localization was addressed in HeLa cells infected at MOI of 1 and incubated without or with IFNα (1000 U/mL, 24 h) before nuclear/cytosol fractionation and immunoblotting (Fig. 3A,B) or immunolabeling of cells (Fig. 3C).

After IFNα stimulation (Fig. 3A,B), Tha virus retained pSTAT1 in the cytoplasm (cytoplasm/nuclear ratio upper than 3). In comparison, mutation of Th2P and Th4M proteins partially restored the capacity of pSTAT1 to translocate to the nucleus in infected cells (pSTAT1 cytoplasm/nuclear ratios of 1.2 and 1.6, respectively). Moreover, pSTAT1 accumulated in the nuclei of Mock and Th2P-4M infected cells compared to Tha-, Th2P- or Th4M-infected cells, (Fig. 3B). These observations suggest that Tha-virus P and M proteins cooperate in interaction with and cytoplasmic retention of pSTAT1. Immunolabeling experiments confirmed those results (Fig. 3C).

pSTAT1 cytoplasmic retention in ThP_265_- or ThP_287_-infected cells was not impaired after IFNα stimulation (Fig. S2), despite the loss of interaction between pSTAT1 and P_265_ or P_287_ (Fig. 2). These findings further corroborate M protein’s ability to compensate for P-protein mutations affecting pSTAT1 interactions.

### P and M proteins cooperate to limit pSTAT1–ISRE-promoter binding

Whether the altered pSTAT1 cytoplasmic retention by P and M proteins affected ISGF3-dependent ISG transactivation was determined using a ChIP assay targeting the ISG15 promoter (Fig. 3D). HeLa cells were infected at MOI of 1 and treated without or with IFNα for 24 h before chromatin co-immunoprecipitation with pSTAT1 or anti-FLAG (control) antibodies. Then, the ISG15 promoter containing an ISRE was quantified by qPCR. pSTAT1-ISRE binding was only observed in the presence of IFNα in Mock-infected cells. Tha virus inhibited pSTAT1-ISRE binding while both Th2P and Th4M permitted a partial binding. In Th2P-4M infected cells, the results did not differ from IFNα treated Mock-infected cells. Therefore, P and M proteins cooperate to prevent pSTAT1/ISGF3 binding to the ISRE promoter, which is consistent with the cooperative activity in cytoplasmic retention of pSTAT1.

### M- and P-protein mutations attenuate RABV virulence

**Figure 4:**
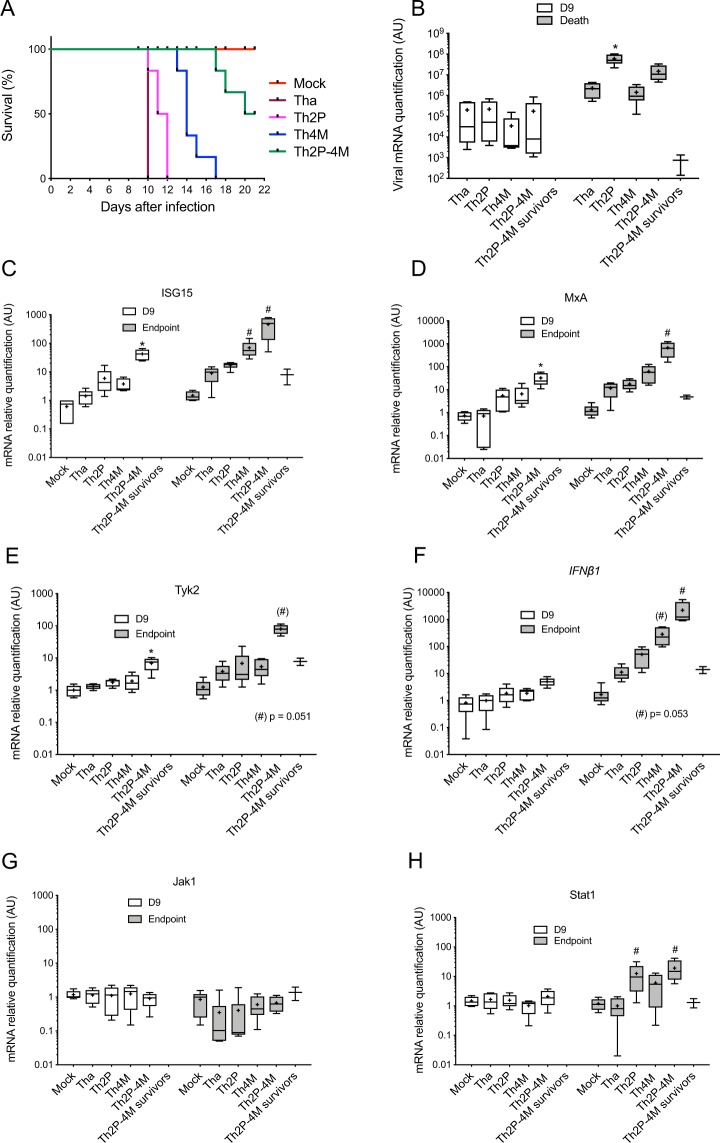
Mutated P and/or M proteins attenuate RABV virulence correlating with differing innate immune-response activation. Three-week-old BALB/c mice (6 per experiment) were infected intramuscularly with 1000 FFUs of Tha, Th2P, Th4M or Th2P-4M RABVs and monitored for 21 days. At the end of the experiment, 3 mice survived when infected with Th2P-4M, named Th2P-4M survivors. (**A**) The mice were sacrificed day 9 post-infection (denoted D9) or when late infection symptoms appeared (endpoint). mRNA, extracted from their brains, was subjected to RT–qPCR to analyze expression of the indicated genes, normalized to the *GAPDH*-reporter gene level in Mock-infected mice. RABV mRNA (**B**), and *ISG15* (**C**), *MxA* (**D**), *IFNβ1* (**E**), *Tyk2* (**F**), *Jak1* (**G**) and *Stat1* (**H**) gene expression was measured. Results show means ± standard deviation (T-bars) expressed in arbitrary units (AU). (NA): non applicable; *p < 0.05 compared to Mock-infected D9 samples and ^#^p < 0.05 compared to Mock-infected End-point samples. Th2P-4Ms*: Th2P-4M survivors.

The pathogenicity of recombinant-RABV was determined in three-week-old female BALB/C mice inoculated intramuscularly with 1000 FFU of Tha, Th2P, Th4M or Th2P-4M. Tha infection caused severe neurological symptoms in all mice by 8 dpi, and all mice succumbed to infection or reached an unresponsive endpoint 10 dpi (Fig. 4A). Th2P virus induced severe neurological symptoms in mice by 9 dpi and death by 10 or 12 dpi, globally comparable to Tha virus. Thus, P-protein mutations did not attenuate pathogenicity in mice. In contrast, Th4M-infected mice showed neurological symptoms and reached the endpoint 3–7 days later than those infected with the Tha virus. Intriguingly, with dual mutated Th2P-4M RABV, symptom onset and endpoint occurred 7–11 days later, and half the mice survived >21 dpi. Therefore, P and M proteins played additive roles in Tha-virus virulence in mice, with M protein having a higher impact.

To confirm the involvement of both P-W265/M287 and M-R77/D100/A104/M110 proteins in the modulation of Jak-Stat signaling *in vivo*, we quantified the transcription of specific ISRE-promoter-dependent genes, such as *MxA* and *ISG*1*5*. Transcription of key genes of the different Jak–Stat-pathway steps (i.e., *IFN*β*1* (activator), *Tyk2* and *Jak*1 (signal transducers) and *Stat*1 (transcription factor), were also analyzed. Total RNAs were extracted from the brains of mice infected by the various RABV mutants, at 9 dpi and at the experimental endpoint. Notably, RABV mRNA was detected in large amounts (>10^6^ RNA copies/μL) in all mouse brains at 9 dpi, with no significant variations, indicating that viral propagation was not perturbed by mutation of P and/or M proteins (Fig. 4B).

However, at the terminal endpoint, the viral load was significantly higher (p < 0.05) in the brain of theTh2P- infected mice than in Tha-infected ones.

Levels of the different transcripts did not differ between Tha- and Mock-infected mice at 9 dpi or at the experimental endpoint. Levels of *ISG15*, *MxA and Tyk2 transcripts* were significantly (p < 0.05) increased in the brains of Th2P-4M-infected mice (*ISG15*, Fig. 4C, *MxA*, Fig. 4D and *Tyk2*, Fig. 4E, white boxes). The same pattern was observed for *MxA* and *Tyk2* transcripts at the experimental endpoint, although they were detected at greater levels in all RABV-infected mice (*MxA*, Fig. 4D and *Tyk2*, Fig. 4E, grey boxes). Concerning *ISG15* transcripts, they were significantly (p < 0.05) increased in the brain of both Th4M and Th2P-4M infected mice (*ISG15*, Fig. 4C, grey boxes). Regarding *IFN*β*1* (Fig. 4E, white boxes), *Jak1* (Fig. 4G, white boxes) and *Stat1* (Fig. 4H, white boxes), no significant effect was observed at 9 dpi between Tha virus and the mutated ones. At the terminal endpoint, *IFN*β*1* transcripts were significantly increased in Th4M- (p = 0.053) and Th2P-4M-infected mice (p < 0.05, Fig. 4E, grey boxes) while *Tyk2* transcripts were only increased in Th2P-4M-infected mice (p = 0.05, Fig. 4F, grey boxes). *Jak1* transcription was not affected by Tha mutants and *Stat1* transcripts were only increased in Th2P and Th2P-4M-infected mice compared to Tha infected ones (Fig. 4H, grey boxes).

The same experiments were run on mice with a different genetic background, C57BL/6, and yielded similar results for *ISG15* and *IFN*β (Fig. S3). Conversely, for *MxA*, *Tyk2* and *Stat1* transcripts, the same trend was observed but the differences were not significant in Th2P-4M-infected mice compared to Tha-infected mice.

These results are also consistent with the quantification of *ISG15*, *MxA*, *IFN*β, *Tyk2*, *Jak1* and *Stat1* in IFNα-stimulated cells (Fig. S4A–F). The correlation between the levels of *ISG15*, *MxA*, *IFN*β, *Tyk2*, *Jak1* and *Stat1* observed and an increased inflammation of the brain in the Th2P-, Th4M-and Th2P-4M-infected mouse as confirmed through histopathological analysis (Fig. S5).

Thus, the data indicated that cooperation between P and M proteins is required for the efficient inhibition by RABV of the innate immune response in mice. In Th4M- and Th2P-infected mice, transcription of ISRE-promoter-dependent genes, such as *MxA* and *ISG15* and of some other key ISG targets of the Jak-Stat-pathway, was still controlled, culminating in 100% mortality. From 9 dpi, Th2P-4M infection induced substantial increases in transcription of these both genes. This can be related to the lower virulence observed at D21 (50% of survival) (Fig. 4).

### P and M proteins cooperate in JAK1 interactions

**Figure 5:**
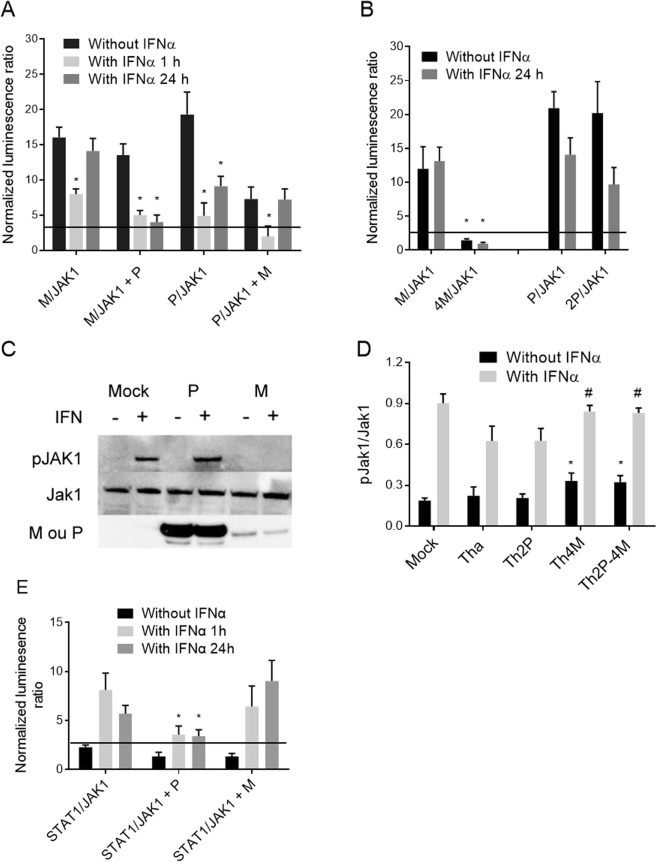
RABV M protein inhibited Jak1 activation and P protein blocked Jak1-Stat1 interaction. Protein complementation assay: wild-type (**A**) or mutated M (Th4M) or P (Th2P) protein (**B**) was co-transfected with JAK1-protein–coding plasmids and stimulated for 1 or 24 h with IFNα or not. Two days post-transfection, the *Gaussia* luciferase activity, whose intensity corresponds to interaction, was determined. P and M proteins were also transfected as third partners in some experiments. The bold horizontal lines represent *Gaussia* significance threshold. Results are expressed as means ± standard deviation (T-bars) of at least four independent experiments. *p < 0.05 compared to unstimulated samples. (**C**) Phosphorylated JAK1 protein (pJAK1), JAK1, and P and M proteins were detected by immunoblotting of cell lysates with specific antibodies. HeLa cells were transfected with wild-type P or M-proteins and stimulated (+) with IFNα (1000 U/mL, 24 h) or not (−). (**D**) pJAK1 ELISA was run on HeLa cells infected with RABV wild-type (Tha) or P- and/or M-mutated proteins (Th2P, Th4M, Th2P-4M) and stimulated or without IFNα. *p < 0.05 compared to Tha-infected samples not stimulated by IFNα and ^#^p < 0.05 compared to IFNα-stimulated Tha-infected samples. Protein complementation assay (**E**): STAT1 and JAK1 proteins were co-transfected and stimulated for 1 or 24 h with IFNα or not. P and M proteins were also co-transfected as third partners in some experiments. Two days post-transfection, *Gaussia* luciferase activity, whose intensity corresponds to interaction, was determined. The bold horizontal lines represent *Gaussia* significance threshold. Results are expressed as means ± standard deviation (T-bars) of at least four independent experiments. *p < 0.05 compared to the corresponding data for STAT1/JAK1 alone.

Next, we investigated other possible interactions between RABV proteins and the Jak–Stat pathway. Previous mass-spectroscopy experiments had shown that M protein is present in JAK1-protein complexes^20^. PCAs performed with M and JAK1 proteins showed that they interact, with or without IFNα treatment (Fig. 5A,B), although 1 h of IFNα stimulation led to fewer NLR signals. By contrast, the 4M protein lost its capacity to interact with JAK1, indicating that the four mutated residues play key role(s) in this interaction (Fig. 5B). Surprisingly, P protein was also found to interact with JAK1 (Fig. 5A). The interaction of P protein or its 2P mutant with JAK1 was conserved with IFNα stimulation or without, despite significantly a lower NLR signal (p < 0.05) after IFN stimulations (for 1 or - hours) (Fig. 5A,B).

In the next step, we investigated whether the P or M protein could interfere with one another’s interaction with JAK1. Co-transfected P protein has no effect on M/JAK1 interactions in the absence of IFN stimulation. However, after 1 or 24 h of IFN stimulation, co-transfected P protein did not abolish M/JAK1 interactions but led to a significantly (p < 0.05) lower NLR signal. As far as M protein is concerned, co-transfected M protein did not abolish P/JAK1 interactions although a lower NLR signal is observed. Most notably, after 1 h of IFN stimulation, co-transfected M protein abolished P/JAK1 interaction (Fig. 5A). However, 24 h of IFN stimulation restored P/JAK1 interactions, despite the presence of M protein.

Thus, the data indicated that P protein can bind to JAK1 and that M protein reduces this P/JAK1 interaction during the early times of IFN stimulation.

### M protein inhibits JAK1 phosphorylation and P protein prevents JAK1-STAT1 interaction

To investigate the effect of M/JAK1 interaction, we monitored JAK1 phosphorylation (pJAK1) in cells, with or without RABV protein expression, by Western blotting. HeLa cells were first transfected with wild-type P or M protein, then incubated with IFNα for 24 h (Fig. 5C). In untransfected cells, JAK1 was phosphorylated only in the presence of IFNα (Fig. 5C). P protein did not impair JAK1 phosphorylation but M protein impaired JAK1 phosphorylation, despite IFNα stimulation (Fig. 5C).

Those findings were confirmed by ELISA on RABV-infected cells which allow us to quantify pJAK1/JAK1 (Fig. 5D). Without IFNα activation, only a minor fraction of JAK1 in Mock-infected cells were phosphorylated (pJAK1/JAK1 = 0.15) compared with IFN-treated Mock-infected cells and neither Tha nor Th2P infection impaired that level of JAK1 phosphorylation. However, mutated Th4M and Th2P-4M increased phosphorylation of JAK1 proteins (pJAK1/JAK1 = 0.3). After IFNα treatment, nearly half of JAK1 proteins in Mock-infected cells was phosphorylated (pJAK1/JAK1 = 0.9), while pJAK1 levels were significantly (p < 0.05) lower in Tha- and Th2P-infected cells (pJAK1/JAK1 = 0.6) (Fig. 5D). Moreover, in Th4M- and Th2P-4M–infected and IFN-treated cells, JAK1 phosphorylation was similar to that of Mock-infected and IFN-treated cells (pJAK1/JAK1 = 0.9). Therefore, through its interaction with JAK1, M protein alone was able to inhibit JAK1 phosphorylation.

To better elucidate the role of P protein on the Jak-Stat pathway, we analyzed JAK1/STAT1 interactions without or with wild-type P protein (Fig. 5E). Upon IFNα stimulation of the Jak-Stat signaling, JAK1/STAT1 complex formation is not blocked by P or M expression (Fig. 5E).

### P and M protein cooperate temporally to fully inhibit the JAK-STAT pathway

**Figure 6:**
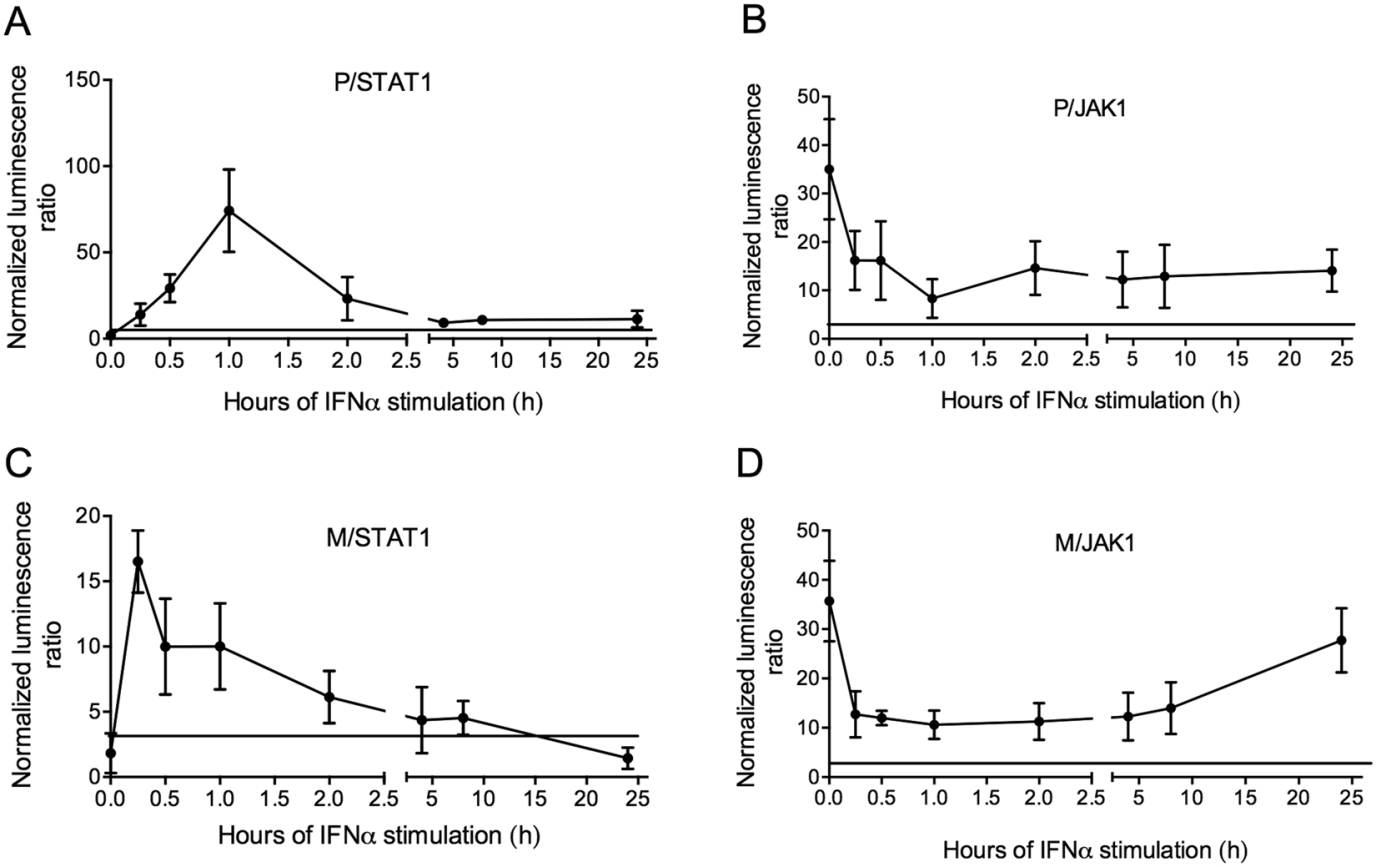
Dynamic interactions between RABV P and/or M proteins and pSTAT1 and Jak1 proteins. Protein complementation assay: P and M proteins were co-transfected with STAT1 or JAK1-coding plasmids. Two days post-transfection, cells were stimulated with IFNα for 15 or 30 min, or 1, 2, 4, 8 or 24 h. *Gaussia* luciferase activity, whose intensity corresponds to interaction, was determined. The bold horizontal lines represent the *Gaussia* significance threshold. Results are expressed as means ± standard deviation (T-bars) of five independent experiments.

All our results showed that the interactions among JAK1, STAT1, P and/or M proteins are modulated after Jak–Stat-pathway activation. Therefore, we followed protein–protein interactions with PCA over 24 h of incubation with IFNα. After an initial sharp M/JAK1-interaction drop, interactions increased from approximately 10 h of stimulation onwards (Fig. 6D). In contrast, M/STAT1 interactions rose during the very early stages of activation of Jak–Stat signaling and rapidly declined thereafter (Fig. 6C). After approximately 10 h of IFNα exposure, M protein no longer interacted with pSTAT1 proteins (Fig. 6C). A correlation was similarly observed between P-protein interactions with STAT1 or JAK1. After IFNα activation, P/JAK1 interactions rapidly diminished (Fig. 6B), and this low level of interaction persisted throughout the post-IFN-stimulation period (35 h) and never returned the initial maximum level. P/pSTAT1 NLR values showed an inverse relationship to P/JAK1 NLR values. P proteins always interacted with pSTAT1 after IFNα, with NLR values peaking after 1 h of IFNα stimulation and persisting over 24 h (Fig. 6A)

To better understand the dynamics of interactions among M, P, STAT1 and JAK1, protein/protein interactions were recorded with PCAs 24 h post-transfection in cells over 1 or 24 h of IFNα stimulation (Fig. S6). Our results indicate that P and M proteins interact together, regardless of JAK/STAT-pathway status activation by IFNα. Overexpression of STAT1 (but not JAK1) impaired P/M interactions after IFNα stimulation in the presence of Stat1 but not in the presence of Jak1.

Taken together, all our findings suggest that P and M proteins cooperated inversely to inhibit Jak and Stat signaling in a time-sensitive manner: pSTAT1/RABV-protein interactions might be essential at infection onset, whereas those with JAK1 might be required during late infection stages.

## Discussion

Type-I IFNs and Jak-Stat signaling constitute a pillar of the innate immune response, triggering ISG production and establishing an antiviral environment inside infected and in neighboring cells^24^. As a consequence, developing escape strategies to hijack and/or inhibit this pathway constitutes a milestone for many viruses in establishing efficient infections.

The P-protein interaction with STAT proteins has been extensively described^16–18^, yet to our knowledge, the interaction of RABV P-protein with JAK1 is described here for the first time. Interestingly, the positions 265 and 287 necessary for P/STAT1 interaction were not involved in P/JAK1 interaction, which suggests that P/JAK interaction is independent of STAT1 and occurs via different mechanisms. Surprisingly, while P-protein interaction with JAK1 interfered with JAK1/STAT1 interaction, it apparently did not affect JAK1 or STAT1 phosphorylation after 24 h of IFNα stimulation.

**Figure 7:**
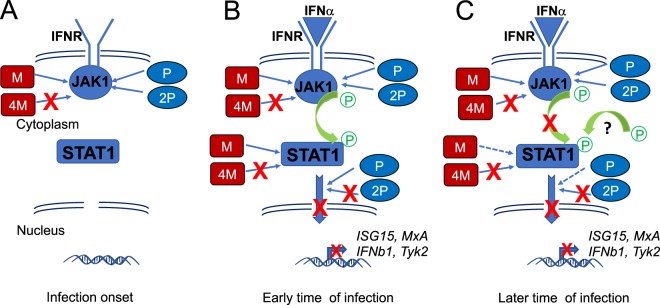
Model of the cooperation between M and P proteins to inhibit different Jak-Stat-pathway steps. (**A**) At infection onset, before the IFN response is triggered and IFNα binds to its receptor (IFNR), JAK1 and STAT1 are not phosphorylated. Then, both RABV M (red) and P (purple) proteins can interact with JAK1, although M-STAT is favoured. Only the mutant 2P (pink) but not 4M (orange) interact with JAK1 (phosphorylated or not). (**B**) In the presence of IFNα, JAK1 is phosphorylated which lead to the phosphorylation of STAT1 and translocation in the nucleus of STAT phosphorylated dimers and finally activation of ISRE promotors and increase of transcription of corresponding genes (ex: ISG15, MxA, IFNβ1 and Tyk2). This is inhibited in the context of a RABV infection by both M and P proteins. Neither the mutant 2P nor 4M are able to interact with STAT1 (phosphorylated or not). Less than 30 min after IFN stimulation (1), the M protein blocked JAK1 phosphorylation and then STAT1 phosphorylation. 30 min after the initial stimulation (2), P destabilises M-JAK1 and the M protein switches towards pSTAT1 interaction, which then enhances the capacity of P protein to also switch towards pSTAT1 interaction and inhibit the translocation of phosphorylated STAT dimers. (**C**) Several hours later, P and M proteins tend to interact preferentially with JAK1, although the initially high P/JAK1 levels were never again reached.

Herein, we found that M protein interference with cell signaling is not only affecting pathways regulating type-I IFN expression^19,20,22^ but also type-I IFN response. Thus, we propose a model (Fig. 7) where M protein cooperates with P protein to control the Jak-Stat signaling pathway. M protein interacted dynamically with JAK1 to partially prevent the JAK1 phosphorylation and, hence, its activation. JAK1 phosphorylation is mandatory for transduction of the IFNAR signal to its downstream targets, STAT proteins^25^. While the M protein did not affect STAT1 phosphorylation, it was able to participate independently of other viral proteins in: 1) pSTAT1 cytoplasmic retention, 2) restriction of STAT1 access to its target, the ISRE promoter, and 3) control of expression of ISGs. Whether pSTAT1 cytoplasmic retention relates to the observed M/pSTAT1 interaction remains to be investigated. Furthermore, it should be considered that the inhibition of JAK1 phosphorylation might be associated with the regulation of other pathways downstream from JAK1, such as mitogen-activated protein kinase (MAPK) or serine/threonine kinase-1 (AKT)^26^.

Residues 77, -100, -104 and -110 of M protein were initially identified as important to M protein interaction with RelAp43 by mutagenesis^22^, and subsequently involved in the interaction with TNF-inducible zinc finger protein A20-binding protein (ABIN2) and mitogen-activated MAP3K tumor-progression locus-2 (TPL2), both proteins complexed with RelAp43 and a second NF-κB protein, p105^20^. In the present study, these four positions also proved to be important for interactions between the M protein and JAK1 and/or STAT1, supporting the role of the four residues and their surrounding regions in M-protein functions. Although mutation of the M protein had only a mild effect on virus replication, it strongly impacted RABV escape from the immune response. If the global structure of the protein remains intact and preserves its vital functions for replication (transcription regulation, viral entry, budding), the four amino-acid mutations impairs M-protein interactions with up to five host proteins involved in NF-κB and JAK-STAT pathways^27–29^.

Interactions between both RABV proteins and JAK1 seem to be essential before IFN induction of signaling and during the later infection stages. These dual interactions could limit signal outbursts early during the infection and enable control of later feedback loops^30^. In contrast, P- and M-protein interactions with pSTAT1 were crucial during the first few hours following the induction of type-I IFN expression. Our study is consistent with the pre-existing model of P/pSTAT1 interactions: P protein and its isoform P2 retain pSTAT1 in the cytoplasm, while the P3 isoform restricts its access to the ISRE promoter in the nucleus and exports it to the cytoplasm^15,16^. In that context, the M protein facilitated P/pSTAT1 interactions, and M- and P-protein cooperation was required to completely inhibit Jak–Stat signaling in field isolates. That a RABV protein evolved to so broadly neutralize the innate immune response means that suppression of multiple pathways of host defenses is required to achieve successful infection.

The results observed *in vivo* are preliminary and can not entirely reflect potential contributions from differences in innate and adaptative immunity. Futher LD50 experiments performed in immunocompetent versus immunocompromised mice will provide a better understanding of the role of these mutations *in vivo*^31^. However, our results corroborate with the fact that depriving RABV of its capacity to interfere with pSTAT1 cytoplasmic retention had a barely noticeable effect on pathogenesis and innate immune responses compared to the wild-type virus. In contrast, depriving RABV of its ability to interfere with the NF-κB and MAPK pathways, as previously described^20^, and JAK1-pSTAT1 signaling significantly impaired virus escape and enhanced host survival. Indeed, because they affect several pathways, M-protein positions 77, 100, 104 and 110 more dramatically impacted RABV escape than P-protein positions 265 and 287, which affected only one viral interference pathway.

In conclusion, our results demonstrate that RABV-M acts as a potent inhibitor of the Jak-Stat pathway in complementation to the P protein and therefore plays a crucial role in shutting down the type-I IFN response and inflammation^19,20,22,32,33^.

## Materials and Methods

### Cell lines and viruses

Human carcinoma epithelial cells (HeLa, ATCC CCL2™) and BSR cells^34^ respectively, are part of the collection of our laboratory and were cultured at 37 °C, 5% CO2, in Dulbecco’s minimal essential medium (DMEM) supplemented with 10% calf serum. Human epithelial kidney cells stably transfected with an ISRE-luciferase reporter gene-37 (STING37) reporter cells kindly provided by Marianne Lucas-Hourani (Unité de Génomique Virale et Vaccination, Virology Department, Institut Pasteur)^35^, were cultured at 37 °C, 5% CO2, DMEM supplemented with 10% calf serum. BSR-T7 cells provided to us by K.K. Conzelmann (Max von Pettenkofer Institute and Gene Center, Munnich)^23^ were cultured in Glasgow medium supplemented with 10% calf serum, tryptose phosphate, nonessential amino acids and geneticin. Human neuroblastoma cells (SK-N-SH ATCC® HTB-11™) were differentiated into neurons in neurobasal medium supplemented with 10% calf serum, glutamax and B19 (Gibco A10508) for 1 week.

Six recombinant viruses from the rabies strain 8743THA (EVAg collection, Ref-SKU: 014V-02106), a street-dog isolate from Thailand^36^, were used: the wild-type virus (Tha), three P-protein mutants (Th2P (P-W265G/M287V), ThP_265_ (P-W265G), ThP_287_ (P-M287V), an M-protein mutant (Th4M (M-R77K/D100A/A104S/M110L)^20,22^ and a mutant bearing mutations on both P- and M-protein (Th2P-4M (P-W265G/M287V and M-R77K/D100A/A104S/M110L).

### Plasmids and sited-directed mutagenesis

All the sequences were amplified by reverse transcription-polymerase chain reaction (RT-PCR) on RNA extracted from Tha-virus–infected HeLa cells using specific primers. PCR products were inserted into the vector of interest using In-Fusion^TM^ HD Cloning Kit (Clontech).

Viral P and M sequences were cloned, respectively, with C-terminal and N-terminal FLAG-tags into the pIRES vector (Clontech, PT3266-5) using *Nhe*I and *Xho*I restriction sites. The complete Tha-virus genome was inserted into pSDI-Flash-HH-SC^37^, as previously described^22^. Plasmids for the protein-complementation assays (PCA) were obtained by cloning M, P, Stat1 and Jak1 sequences into vectors containing the N-terminal (pCMV-KDEL-Glu1) or C-terminal part (pCMV-KDEL-Glu2) of *Gaussia* luciferase, respectively, using *Bst*XI/*Sal*I and *Xho*I/*Sac*II restriction sites^38^. To study the impact of a third protein in PCAs, plasmids coding for M, P, STAT1 or JAK1 protein without FLAG- or HA-tag were used.

Mutations were introduced into the Tha-genome or P- and/or M-protein plasmids using Change-IT^TM^ Multiple Mutation Site-Directed Mutagenesis Kit (Afflymetrix) and specific primers, as described previously^22^.

### Reverse genetics

Recombinant RABVs were rescued, as previously described^22^, by transfection into BSR-T7 cells of the complete genome (2.5 μg), and plasmids N-pTIT (2.5 μg), P-pTIT (1.25 μg) and L-pTIT (1.25 μg). Briefly, 6 days after transfection, supernatants were passaged onto BSR cells and incubated for 5 days. The supernatant was harvested and titrated on BSR cells. To control for the isolation of different RABVs, growth curves were plotted. BSR cells were infected at a multiplicity of infection (MOI) of 0.1 fluorescent focus units (FFUs)/cell. Supernatants were harvested 24-, 48- and 72-h post-infection and titrated on BSR cells. RABV proteins were extracted from the infected cells and subjected to Western-blot analysis.

### Transfection, infection and stimulation

Lipofectamine 2000 (Invitrogen) was used for all transfections at a ratio of 1:2.4 (μg of DNA/μL of Lipofectamine 2000) for HeLa, SK-N-SH and BSR-T7 cells, and 1:3 for HEK-293T cells. HeLa, differentiated SK-N-SH and STING37 cells were infected at an MOI of 1 FFUs/cell and washed 2-h post-infection to remove input virus. Twenty-four hours after infection or transfection, cells were stimulated with 1000 U/mL of IFNα and/or 10 ng/mL of tumor necrosis factor (TNF) for 1–24 h in DMEM without calf serum. Western-blot analyses or immunofluorescence experiments were performed on cell extracts. Supernatants were titrated on BSR cells to control for viral infection.

### Ethics statement

All mice experiments were performed in accordance with guidelines of the European and French guidelines (Directive 86/609/CEE and Decree 87–848 of 19 October 1987) and the Institut Pasteur Safety, Animal Care and Use Committee, and approved by the French Administration (Ministère de l′Enseignement et de la Recherche) under the number O522-02. All animals were handled in strict accordance with good animal practice.

### *In vivo* experiments

Three-week-old BALB/C or C57BL/6 mice were infected by intramuscular injection of 1000 FFUs of the different recombinant RABVs and monitored for 21 days.

In this model, the pathology observed in mice infected by Tha virus can be caraterized in 3 steps: (stage 1) D8, first signs of illness: ruffled fur, slow movement, hind limb ataxia and apathy appearing. (stage 2) D9: worsening of symptoms were visible like monoplegia and hind limb paralysis. (stage 3) D10: mice reached an unresponsive endpoint (humane endpoint) characterized by paralysis of at least two limbs, and finally death.

Two different type of experiments were performed: (i) mice were sacrificed 9 days post-infection (dpi), stage 2, when monoplegia and hind limb paralysis were observed in mice infected with Tha virus and/or (ii) mice were sacrificed when they reached an unresponsive endpoint (humane endpoint, paralysis of at least two limbs) at stage 3. The infection was confirmed by RT–quantitative (q)PCR.

### Immunohistochemistry

After necropsy, mouse brains were removed and immediately fixed in 10% neutral-buffered formalin and embedded in paraffin; 4-µm–thick sections were cut and stained with hematoxylin–eosin. In immunohistochemistry, we assessed 1) microglial cell morphology using a rabbit anti-ionized-calcium binding-adaptor molecule-1 (Iba1) primary antibody (#01919741, Wako Chemical, diluted 1:50), 2) astrocyte morphology using chicken anti-glial fibrillary acidic protein (GFAP) primary antibody (ab4674, Abcam, dilution 1:5000) and 3) RABV detection using the rabbit P49-1 antibody (diluted 1:1000)^39^. All immunohistochemistry procedures were done with the Bond III automat (Leica).

### RT-qPCR

Total RNA was extracted from *in vitro*-cultured cells using RNeasy mini kit (Qiagen, Hilden, Germany). Total mouse-brain RNA was isolated with TRIzol. Using Superscript II (Invitrogen) with Oligo-dT primers (2 pmol, Fermentas), 1.2 μg of RNA were reverse transcribed and transcripts were amplified and analyzed with Power SYBR Green (Qiagen, Hilden, Germany) and 7500 Fast Real-Time PCR System (Applied Biosystems) by 7500 SDS software version 2 (Applied Biosystems). Commercial primers (QuantiTect Primer Assay, Qiagen, Hilden, Germany) were used for the following human and mouse genes: *Trim25*, *ISG15*, *Stat1*, *NF-*κ*B1*, *Jak1*, *Tyk2* and *IFN*β*1*. The following primers were used for *MxA*: 5′-ATAGACCTTCCTGGCATAACC-3′ and 5′-CTTCAGTTCCVTTTGCCCACCA-3′. The relative amount of each target mRNA was normalized to the glyceraldehyde-3-phosphate dehydrogenase (*GAPDH*) level in each sample. Commercial primers (QuantiTecy Primer Assay, Qiagen, Hilden, Germany) were used for the following mouse genes: *Trim25*, *ISG15*, *Stat1*, *NF-*κ*B1*, *Jak1*, *Tyk2* and *IFN*β*1*.

### Protein extraction and western blot

Transfected or infected cells were washed with phosphate-buffered saline (PBS). RIPA Lysis Buffer System (Santa Cruz Biotechnology) and the Nuclear, Cytosol Fractionation Kit (CliniScience), respectively, were used for protein extraction, and nuclear and cytoplasmic fractionation. Western-blot were performed with NuPAGE 10% Bis–Tris Gels (Invitrogen) and the iBlot transfer system (Invitrogen). To detect RABV P and M proteins, anti-M (186.20) and anti-P (49.11) mouse antibodies (diluted 1:3000) were used^39^. Phosphorylated STAT1 was detected with a phosphotyrosine-specific rabbit antibody (diluted 1:1000) recognizing phospho-Y701 (Cell signaling, S8D6). Anti-α-tubulin (Sigma, T5168) and anti-lamin (Abcam, 8984) mouse antibodies (diluted 1:1000) served as loading and fractionation controls. Mouse and rabbit antibodies were revealed with horseradish-peroxidase–conjugated secondary antibody (GE Healthcare; diluted to 1:1000). Membrane saturation and all antibody dilutions were made in PBS–Tween 0.1% and 5% nonfat dry milk. Blots were revealed by chemiluminescence on a LA500 blot revealer (Amersham), with LA500 software for blot analyses.

### ELISA

ELISA assays were performed using “RayBio phospho-JAK1 and total JAK1 ELISA” kit, (RayBiotech, Inc., Norcross, GA, USA) according to the manufacturer recommendation. ELISA was run on HeLa cells infected with RABV wild-type (Tha) or P- and/or M-mutated proteins (Th2P, Th4M, Th2P-4M) and stimulated or not with IFNα.

### Immunofluorescence

Infected cells were washed with PBS, fixed with cold methanol and permeabilized with acetone. Immunofluorescence experiments used the same primary antibodies as for Western blots, and secondary conjugated anti-rabbit DyLight 405 (Jackson ImmunoResearch) and conjugated anti-mouse DyLight 549 (Jackson ImmunoResearch) antibodies (diluted 1:1000). Saturation and all antibody dilutions were done in PBS with 10% of calf serum. DAPI was used to stain the nucleus blue (ProlonGold with DAPI). Analyses were done on the Zeiss ApoTome system.

### PCA/luciferase assay

HEK-293T cells were seeded onto 96-well plates and, 8 h later, transfected with 100 ng of Glu1 and Glu2 chimeric constructs, and 5 μg of firefly-luciferase DNA. *Gaussia* and firefly luciferase activities were measured 48-h post-transfection on the GloMax multi-detection system (Promega) using the *Renilla* and firefl*y* Luciferase Assay Systems (Promega), respectively. IFNα (1000 U/mL) was added to cell cultures, 15 and 30 min, and 1, 2, 4, 8 or 24 h before the luciferase readout. *Gaussia*-luciferase activity was normalized to firefly luciferase activity in order to evaluate transfection efficiency. Protein–protein interaction levels are expressed as normalized luminescence ratios (NLRs), according to the following formula, as described previously^40^:

$${\rm{NLR}}=({\rm{Glu}}1{\rm{A}}+{\rm{Glu}}2{\rm{B}})/[({\rm{Glu}}1{\rm{A}}+{\rm{Glu}}2)+({\rm{Glu}}1+{\rm{Glu}}2{\rm{B}})]$$

Glu1A and Glu2B are chimeric proteins, and Glu1 and Glu2 empty vectors. The threshold for positive interaction between two partners was determined for an NLR > 3.5^40^.

### Chromatin immunoprecipitation (ChIP) assay

ChIP assays were performed as previously described^41^. Briefly, after infection and IFN stimulation, cells were incubated for 15 min in culture medium containing 1% formaldehyde, washed with PBS and collected by scraping in 5 mL of buffer (100 mM Tris–HCl [pH 9.4], 10 mM dithiothreitol). Cells were lysed in lysis buffer (0.25% Triton X–100, 0.5% NP-40, 10 mM EDTA, 0.5 mM EGTA, 10 mM Tris [pH 8.0]) supplemented with protease and phosphatase-inhibitor cocktail (Complete; Roche Molecular Biochemicals). Cell lysates were then sonicated to yield 150-bp chromatin fragments, as determined by Agarose-gel electrophoresis. The sonicated protein extracts were diluted 1:10 in dilution buffer (0.01% SDS, 1.1% Triton X–100, 1.2 mM EDTA, 16.7 mM Tris–HCl [pH 8.1], 150 mM NaCl) and then precleared with 50 μL of protein G–Sepharose beads for 3 h at 4 °C. Proteins were immunoprecipitated with anti-human pStat1 and anti-FLAG, overnight at 4 °C. Then, the beads were washed extensively, and cross-links were broken by incubation in elution buffer (1% SDS, 0.1 M NaHCO_3_) for 15 h at 65 °C. DNA was extracted with phenol–chloroform, ethanol precipitated, and dissolved in 50 μL of H_2_O. The input and precipitated DNA were analyzed by real-time PCR using primers encompassing the ISRE-binding site on the ISG15 promoter (forward: 5′-CTCCTCCCTCCCTGAAGCT-3′; reverse: 5′-CGGTTGAGTTTCGTTTCTTGCA-3′. qPCR used Applied Biosystems PRISM 7900HT in triplicate with Hotstar PCR master mix (Qiagen, Hilden, Germany), according to the latter manufacturer’s instructions. PCR consisted of 40 cycles of 95 °C for 15 s and 55 °C for 30 s.

### Statistical analyses

All analyses were computed with GraphPad Prism software. Results are expressed as means ± standard deviation (error bars) of at least triplicate samples. Statistical significance was assessed by ANOVA or Student’s t-tests and defined as p < 0.05.

## Supplementary information

Supplementaries

Supplemental data set_Blot
