# Supplementary material for: Lyssavirus matrix protein cooperates with phosphoprotein to modulate the Jak-Stat pathway
Source: Sci Rep. 2019 Aug 21;9:12171. doi: 10.1038/s41598-019-48507-4 (PMC6704159; doi:10.1038/s41598-019-48507-4)
Supplement: Supplementary file 1 — Supplementaries [file 41598_2019_48507_MOESM1_ESM.pdf]

Lyssavirus matrix protein cooperates with phosphoprotein to modulate the Jak-Stat pathway

Florian Sonthonnax[1,2], Benoît Besson[1,2], Emilie Bonnaud[1], Grégory Jouvion[3], David Merino[1], Florence Larrous[1]*, Hervé Bourhy[1]

Supplementary information

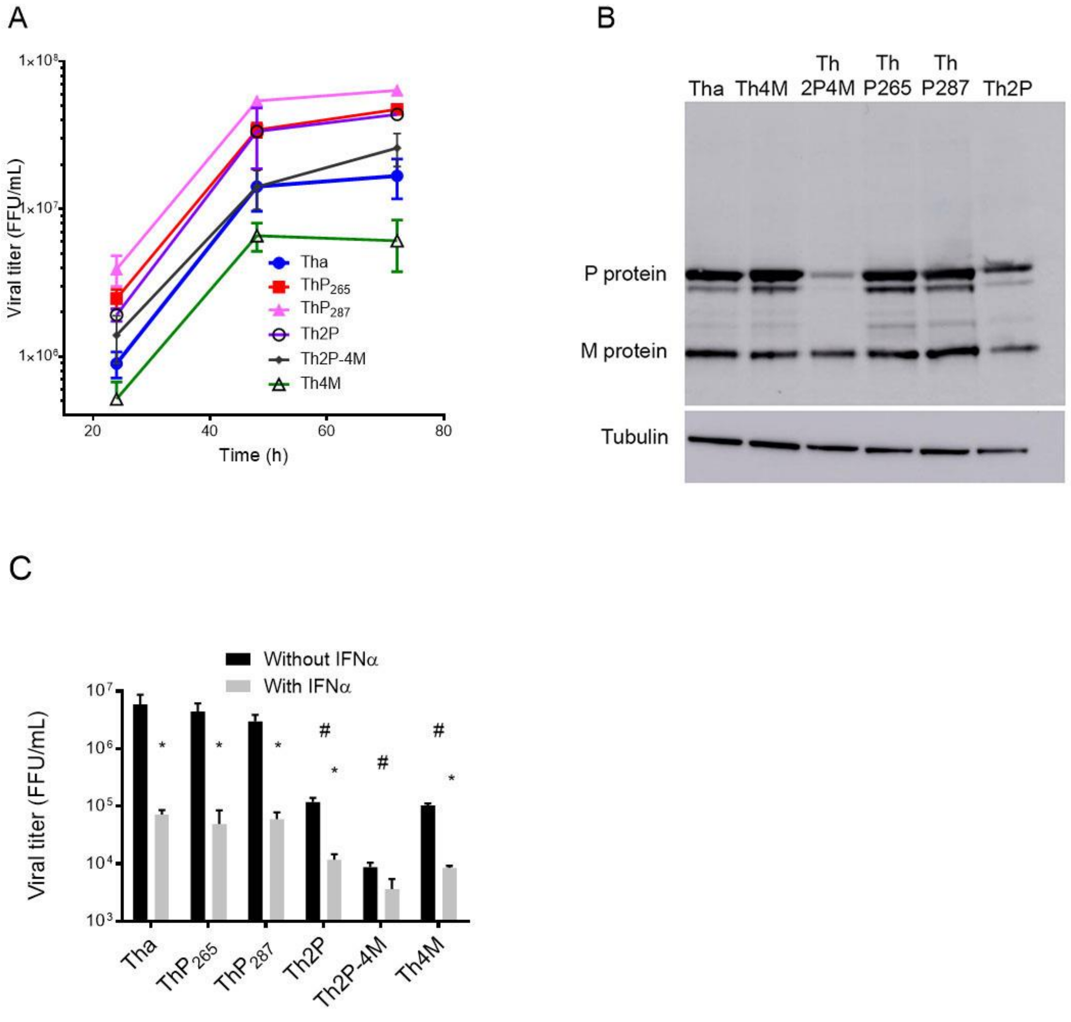

Fig. S1 Characterization of mutated recombinant rabies viruses (RABVs). (A) BSR-T7 cells, which lack an IFN response were infected with wild-type virus (Tha) or virus containing mutated P- and/or M protein (ThP_{265}, ThP_{287}, Th2P, Th4M, Th2P-4M). Virus titers were measured in the supernatants at 24, 48 and 72 h post-infection. Results are expressed as the means ± standard deviation (T-bars) of three independent experiments. (B) BSR-T7 cells, were infected with wild-type virus (Tha) or virus containing mutated P- and/or M protein (ThP_{265}, ThP_{287}, Th2P, Th4M, Th2P-4M), and P and M viral proteins were detected by Western blotting

48 h post-infection. Tubulin detection served as a loading control. (C) HeLa cells, which have a competent innate immune response, were infected with wild-type (Tha)- or P- and/or M-mutated protein (Th2P, Th4M, Th2P-4M)-viruses. IFNα (1000 U/mL, 24 h) (grey) was added or not (black) 24-h post-infection. Virus titers were measured in the supernatants 48 h post-infection. Results are expressed as the means ± standard deviation (T-bars) of three independent experiments. * $p<0.05$ compared to Tha-infected samples non-stimulated by IFNα and # $p<0.05$ compared to IFNα-stimulated Tha-infected samples

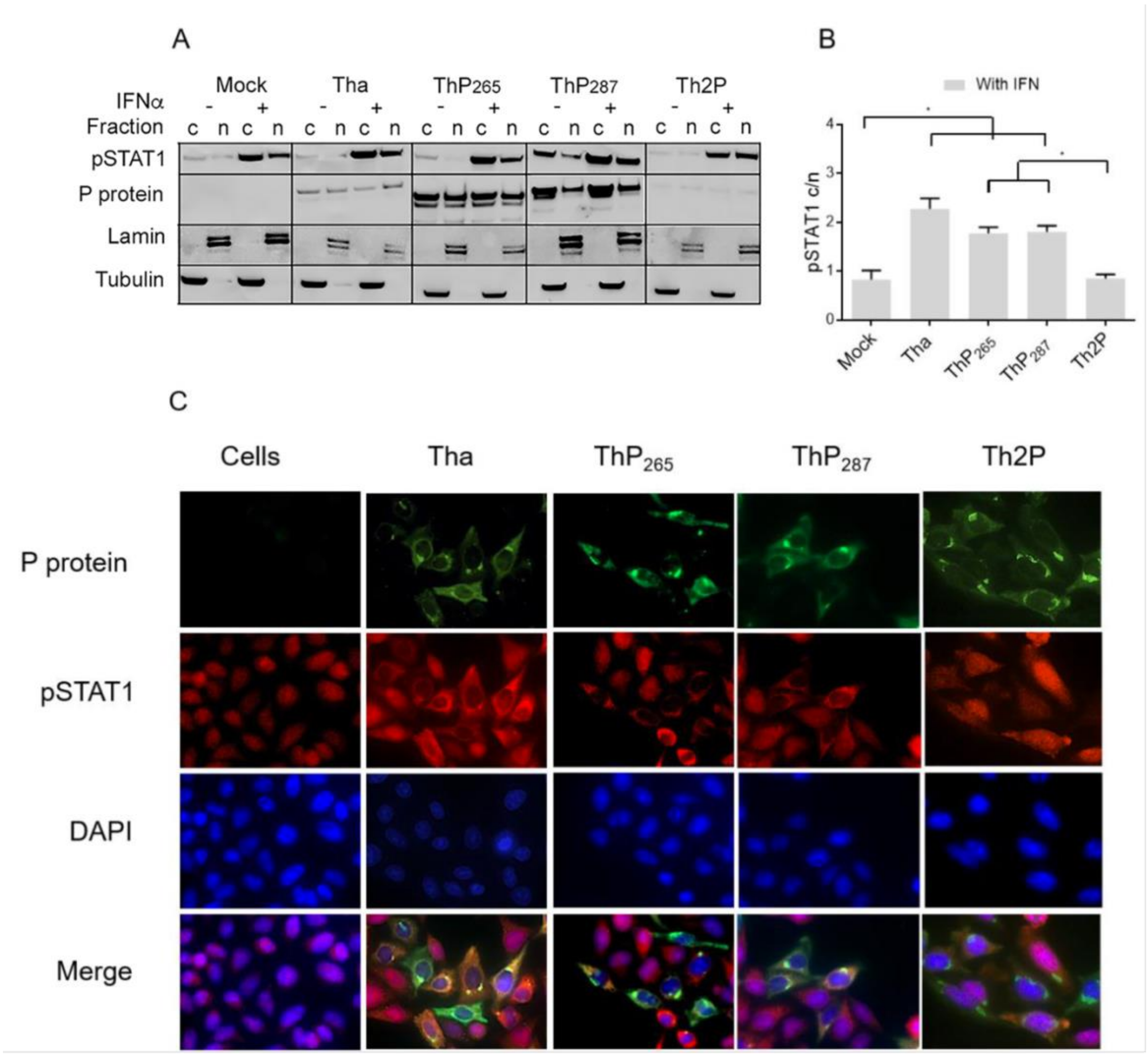

Fig. S2 Phosphoprotein (P) involvement in pSTAT1 cytoplasmic retention. (A) After cytoplasmic and nuclear fractionation of HeLa cells infected with wild-type (Tha) or P-mutated (ThP_{265}, ThP_{287}, Th2P) RABVs, stimulated with IFNα (1000 U/mL, 24 h) or not, P and pSTAT1 proteins were immunolabeled with specific antibodies. Lamin and tubulin served as controls. c: cytoplasmic fraction, n: nuclear fraction. (B) pSTAT1 cytoplasm/nuclear quantification based on three independent experiments, expressed as means ± standard deviation (T-bars). * $p<0.05$. (C) Specific antibody immunolabeling of pSTAT1 and P proteins in cells infected with the different recombinant RABVs. DAPI stained the nuclei blue. Images of non-, Tha- and Th2P-4M-infected cells are identical to the respective ones presented in Figure 3.

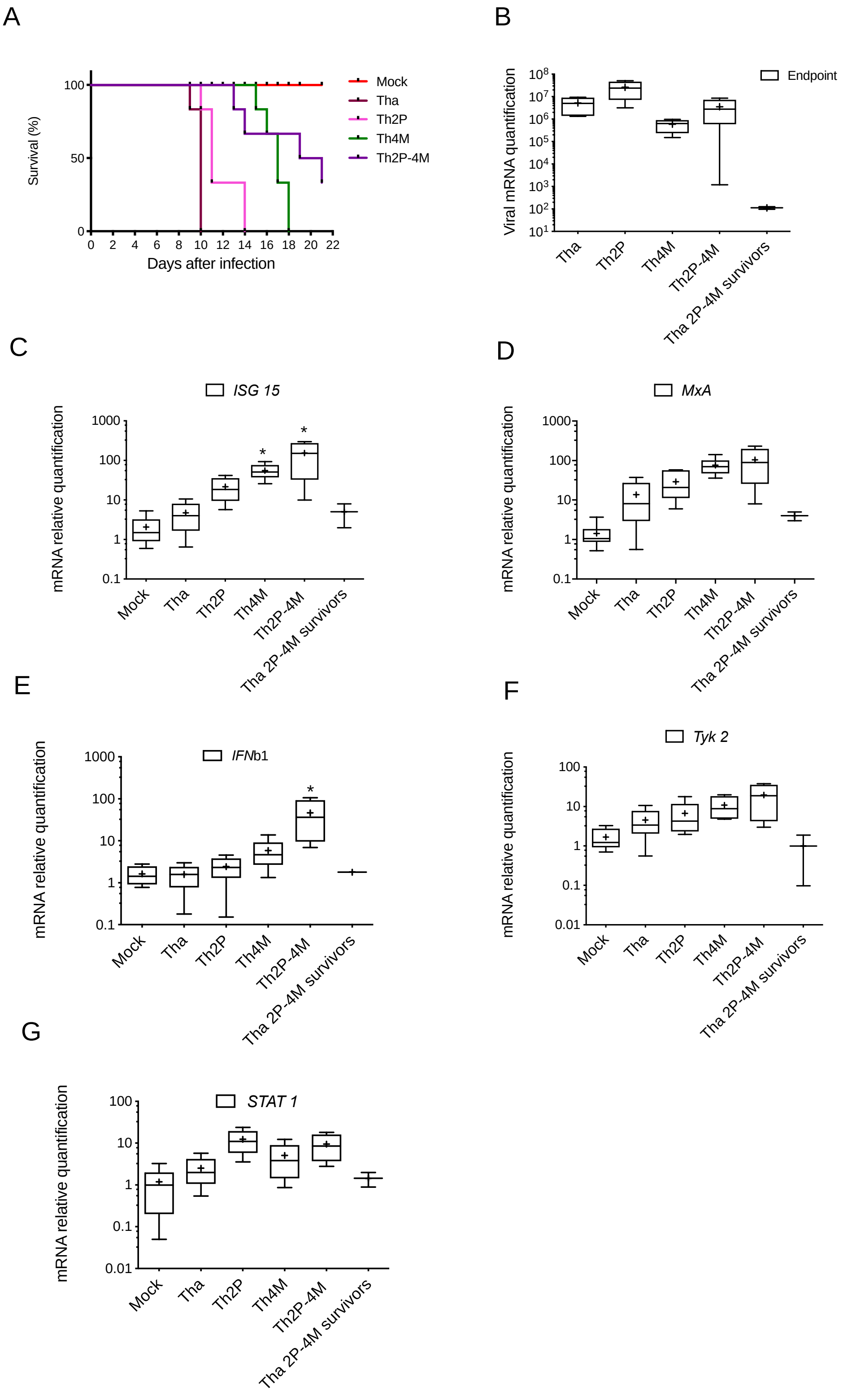

Fig. S3 P- and/or M-protein mutations attenuated RABV virulence in a fashion corresponding to innate immune-response activation. Three-week-old C57BL/6 mice (6 per experiment) were infected intramuscularly with 1000 FFUs of Tha, Th2P, Th4M and/or Th2P-4M viruses and monitored for 21 days. At the end of the experiment, 2 mice survived when infected with Th2P-4M and were named Th2P-4M survivors (A). The mice were

sacrificed when late infection symptoms appeared. mRNA, extracted from their brains, was subjected to RT–qPCR to analyze gene expression, normalized to *GAPDH*-reporter–gene levels of non-infected mice (Mock). RABV P-protein mRNA levels (B), and *ISG15* (C), *MxA* (D), *IFNβ1* (E), *Tyk2* (F) and *Stat1* (G) gene expression is shown. The results are presented in "boxes" delimited by the minimum and maximum values of the replicates ± standard deviation (T-bars) expressed in arbitrary units (AU). The inner line corresponds to the median value and the cross to the mean. * $p<0.05$ compared to Tha-infected End-point samples.

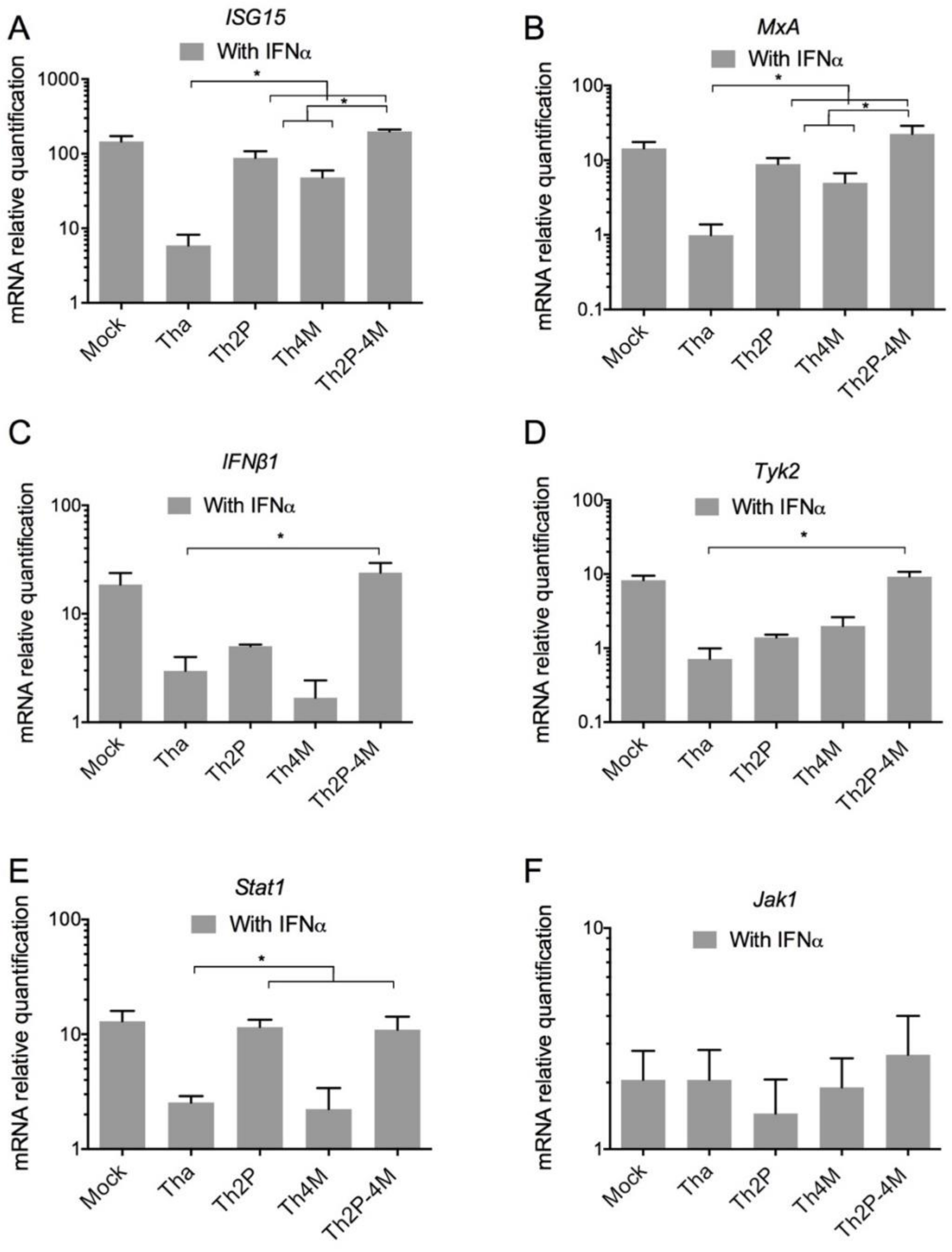

Fig. S4 RABV P and/or M proteins modulate IFN activated gene transcription. HeLa cells were infected with wild-type (Tha) or P- and/or M-mutated–protein (Th2P, Th4M, Th2P-4M) RABVs. After 24 h of infection, IFNα (1000 U/mL, 24 h) was added. mRNA was extracted from HeLa cells. Expressions of the following genes: *ISG15* (A), *MxA* (B), *IFNb1* (C), *Tyk2* (D), *Jak1* (E) and *Stat1* (F) were analyzed by RT–qPCR and normalized to the *GAPDH*- house-keeping-gene level in Mock-infected unstimulated cells. Results are means ± standard deviation (T-bars) of five independent experiments. * $p<0.05$.

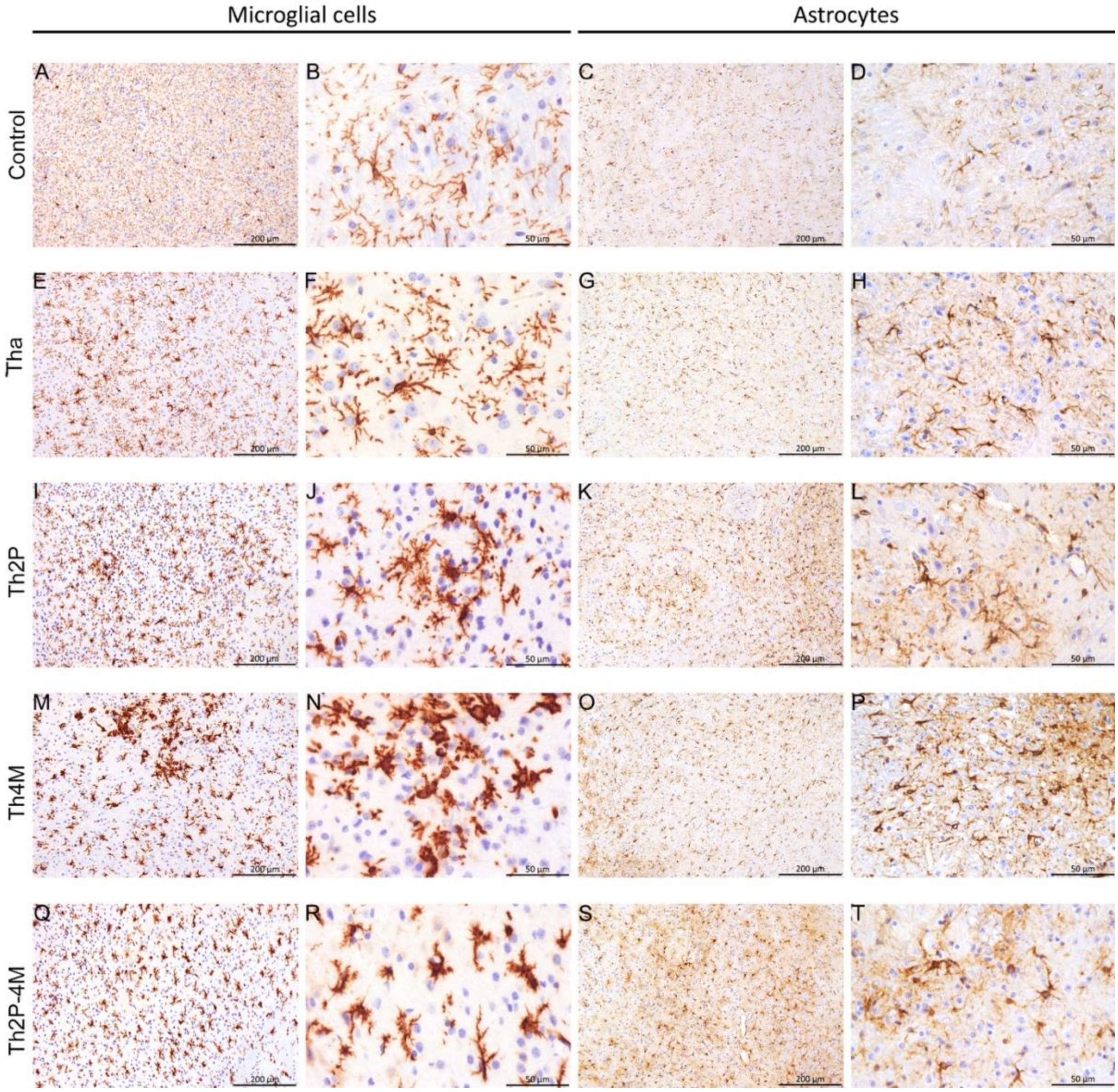

Fig. S5 Analysis of microglial cell reactivity and astrocyte activation. Microglial cell reactivity and astrocyte activation were analyzed by immunohistochemical labeling with anti-Iba1 and -GFAP antibodies, respectively. Control mice (A–D) had no histological lesions. Wild-type Tha-virus–infected mice had encephalitis, characterized by activation of microglial cells (E, F) and astrocytes (G, H). Mice infected by Th2P, Th4M or Th2P-4M RABVs had also signs of encephalitis (I-L, M-P, Q-T, respectively) with activations of microglial cells and astrocytes of various severity. However, more severe lesions were seen with Th4M and Th2P RABVs.

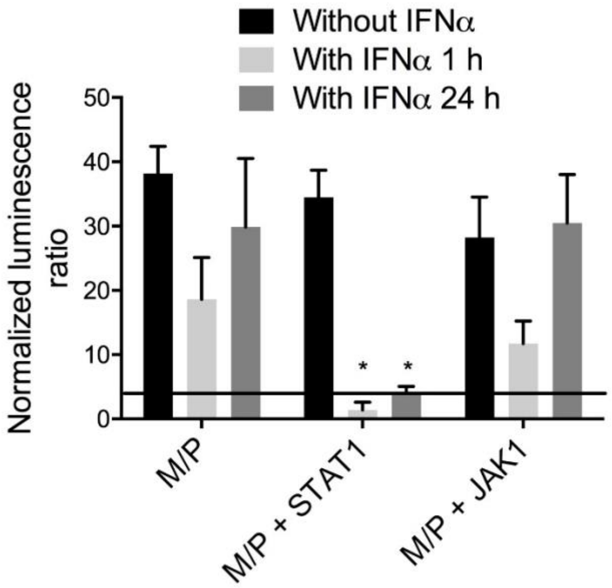

Fig. S6 Interactions between RABV P and M proteins and STAT1 or JAK1. Protein complementation assay: P and M (4M) proteins were co-transfected with STAT1- or JAK1-protein–coding plasmids and stimulated for 1 or 24 h with IFNα or not. Two days post-transfection, the *Gaussia* luciferase activity, whose intensity corresponds to interaction, was determined. The bold horizontal lines represent the *Gaussia* significance threshold. Results are expressed as means ± standard deviation (T-bars) of at least four independent experiments. * $p<0.05$ compared to unstimulated samples.
