# Supplementary material for: Lyssavirus matrix protein cooperates with phosphoprotein to modulate the Jak-Stat pathway
Source: Sci Rep. 2019 Aug 21;9:12171. doi: 10.1038/s41598-019-48507-4 (PMC6704159; doi:10.1038/s41598-019-48507-4)
Supplement: Supplementary file 2 — Supplemental data set_Blot [file 41598_2019_48507_MOESM2_ESM.pdf]

Lyssavirus matrix protein cooperates with phosphoprotein to modulate the Jak-Stat pathway

Florian Sonthonnax[1,2], Benoît Besson[1,2], Emilie Bonnaud[1], Grégory Jouvion[3], David Merino[1], Florence Larrous[1]*, Hervé Bourhy[1]

Supplemental data set

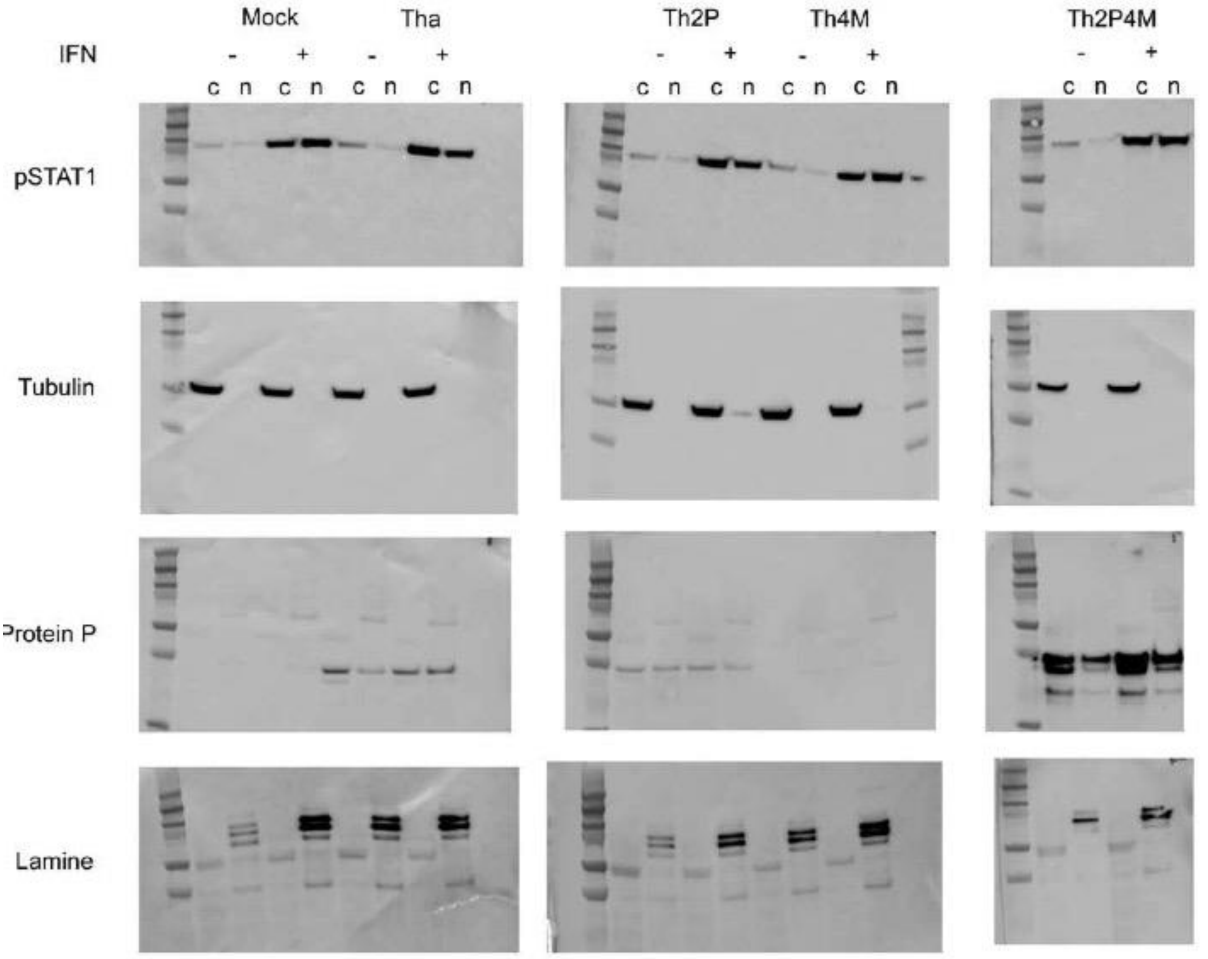

Blots used for Figure 3A

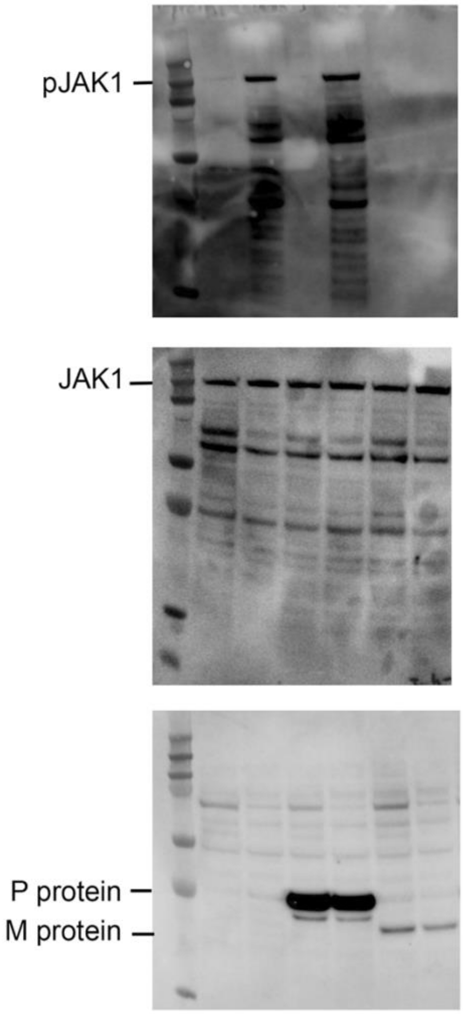

Blots used for Figure 5

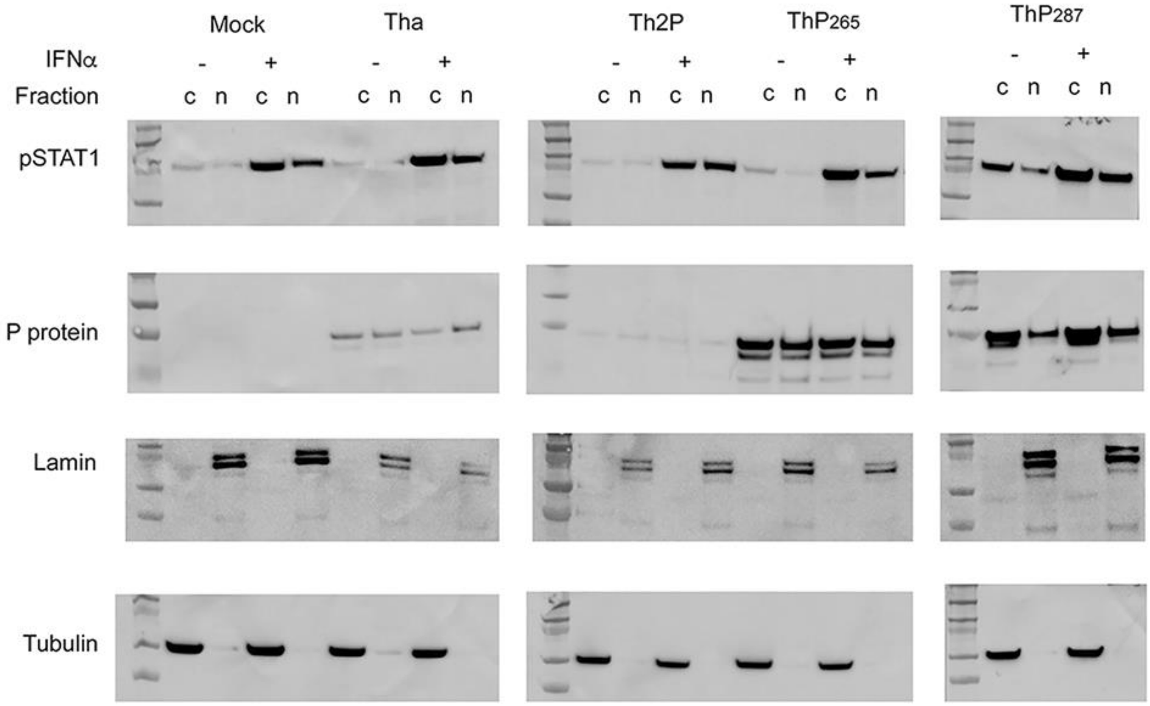

Blots used for Figure S2A
